# Revealing the roles of glycosphingolipid metabolism pathway in the development of keloid: a conjoint analysis of single-cell and machine learning

**DOI:** 10.3389/fimmu.2023.1139775

**Published:** 2023-04-24

**Authors:** Binyu Song, Yu Zheng, Hao Chi, Yuhan Zhu, Zhiwei Cui, Lin Chen, Guo Chen, Botao Gao, Yichen Du, Zhou Yu, Baoqiang Song

**Affiliations:** ^1^Department of Plastic Surgery, Xijing Hospital, Fourth Military Medical University, Xi’an, Shaanxi, China; ^2^Hospital for Skin Disease (Institute of Dermatology), Chinese Academy of Medical Sciences and Peking Union Medical College, Nanjing, Jiangsu, China; ^3^Clinical Medical College, Southwest Medical University, Luzhou, China

**Keywords:** glycosphingolipid, keloid, single cell, machine learning, immune

## Abstract

Keloid is a pathological scar formed by abnormal wound healing, characterized by the persistence of local inflammation and excessive collagen deposition, where the intensity of inflammation is positively correlated with the size of the scar formation. The pathophysiological mechanisms underlying keloid formation are unclear, and keloid remains a therapeutic challenge in clinical practice. This study is the first to investigate the role of glycosphingolipid (GSL) metabolism pathway in the development of keloid. Single cell sequencing and microarray data were applied to systematically analyze and screen the glycosphingolipid metabolism related genes using differential gene analysis and machine learning algorithms (random forest and support vector machine), and a set of genes, including ARSA,GBA2,SUMF2,GLTP,GALC and HEXB, were finally identified, for which keloid diagnostic model was constructed and immune infiltration profiles were analyzed, demonstrating that this set of genes could serve as a new therapeutic target for keloid. Further unsupervised clustering was performed by using expression profiles of glycosphingolipid metabolism genes to discover keloid subgroups, immune cells, inflammatory factor differences and the main pathways of enrichment between different subgroups were calculated. The single-cell resolution transcriptome landscape concentrated on fibroblasts. By calculating the activity of the GSL metabolism pathway for each fibroblast, we investigated the activity changes of GSL metabolism pathway in fibroblasts using pseudotime trajectory analysis and found that the increased activity of the GSL metabolism pathway was associated with fibroblast differentiation. Subsequent analysis of the cellular communication network revealed the existence of a fibroblast-centered communication regulatory network in keloids and that the activity of the GSL metabolism pathway in fibroblasts has an impact on cellular communication. This contributes to the further understanding of the pathogenesis of keloids. Overall, we provide new insights into the pathophysiological mechanisms of keloids, and our results may provide new ideas for the diagnosis and treatment of keloids.

## Introduction

Keloids are usually pathological scars resulting from abnormal repair of injured skin tissues and are clinically manifested by scar growth beyond the trauma (1, 2). Immune cells and inflammatory factors also play an important role in the development of keloid treatment (3). Keloids are a considerable clinical challenge for physicians, given their persistent growth, high recurrence rate following excision, and the substantial physical and psychological burden they impose on patients. Keloids can cause significant cosmetic and functional impairments, leading to pronounced emotional distress. Consequently, healthcare professionals strive to effectively manage keloids to enhance patients’ quality of life and alleviate their suffering (4). Common clinical treatments for keloid include medication, surgical excision, laser treatment, etc. (5). Keloids have a high recurrence rate, making existing treatments unsatisfactory. Understanding the underlying pathogenesis can lead to the development of new treatments to improve outcomes.Because keloids are characterized by increased fibroblast proliferation and a large excess of ECM components, research on keloids has focused on the involvement of fibroblasts in the development of these lesions (2).

Sphingolipid (SL) is an important class of lipids in eukaryotes. Research into their metabolic regulation in dermatology has potential implications for the development of new therapeutic targets (6, 7). SL metabolism can be involved in maintaining the skin barrier and regulating cellular processes with exerting important biological roles in the skin (8, 9). Recent research has revealed that dermal fibroblasts with different phenotypic functions have different lipid status composition, and SL was shown to be the main markers of different lipid composition status. More importantly, SL harbors the capacity to control the heterogeneity of dermal fibroblasts (7). Glycosphingolipids (GSL, sphingolipids with one or more sugars attached) is a subtype of SL. GSL metabolic reprogramming has also been shown to be an integral part of cell development, and the heterogeneity of GSL determines the specific developmental patterns of cellular tissues (10). In contrast to SL, GSL metabolism has been less studied in skin diseases and even less studied in keloids.

The advent of single-cell RNA sequencing (scRNA-seq) technology provides unprecedented molecular information and serves as one of the most important methodological advances and breakthrough technologies that allow us to systematically decipher the cellular heterogeneity and complexity of different tissues (11–13). Thus, exploring fibroblast heterogeneity, cell fate and intercellular communication in keloids with unprecedented single-cell resolution has become a reality.

In this study, we combined keloid microarray datasets and scRNA-seq to comprehensively analyze the potential mechanisms of GSL metabolism pathway in keloids and the roles they play in keloid development and treatment, deepening our understanding of new mechanisms underlying keloid and providing a theoretical basis for subsequent treatment of keloid patients with improved prognosis.

## Materials and methods

### Data processing

Three keloid microarray datasets (GSE7890, GSE145725, GSE44270) and one keloid single-cell transcriptome sequencing dataset (GSE163973) were downloaded from the publicly available Gene Expression Omnibus (GEO) database. Among them, 5 keloid samples and 5 normal samples were from GSE7890, 9 keloid samples and 10 normal samples were from GSE145725, and 9 keloid samples and 7 normal samples were from GSE44270. We normalized the microarray datasets and integrated them using a common set of annotated genes. Batch effects removal was performed by the combat function in the “sva” R package and the integrated expression data was log2 transformed.

### Screening hub genes based on differential expression and machine learning algorithms

This study contained 46 glycosphingolipid metabolism-related genes (GSLMRGs) from the Reactome database (**Supplementary Table S1**). We performed differentially expressed gene analysis of the 46 GSLMRGs in the integrated dataset by the “limma” R package and obtained 9 differential genes (p-value<0.05) and visualized the differential genes by heatmap. We applied two machine learning algorithms to predict significant GSLMRGs. Support vector machine (SVM) is a machine learning technique widely used for classification and regression analysis, and support vector machine-recursive feature (SVM-RFE) algorithm was used in the “caret” R package to screen out significant diagnostic candidates among 46 GSLMRGs. Random forest is a popular classifier and is widely used in medical applications. We use the “randomforest” R package to predict key candidate genes. We took the intersection of the top10 genes predicted by each of the two machine learning algorithms and screened out 6 of the DEGs as the candidate GSLMRGs.

### Diagnostic model building and evaluation

We constructed the diagnostic model by multi-factor logistic regression algorithm using the six candidate GSLMRGs by application of the integrated dataset as the training dataset, and plotted the ROC curve and calculated the area under the curve (AUC) to evaluate the prediction results. The bootstrap analysis was replicated on 1000 different samples of the same sample size drawn with replacements from the original samples. The training samples were regenerated, and the model was reconstructed. And the bootstrap algorithm was used to evaluate the accuracy of the diagnostic model we built. The nomogram was built in expectation of making the correct diagnosis.

### Immune cell infiltration analysis

A pearson correlation analysis was carried out to reveal the association among the 6 GSLMRGs based on the RNA expression data by corrplot R package. We used the CIBERSORT and EPIC algorithm by the “IOBR” R package to calculate the degree of immune cell infiltration in the samples (34276676) and correlated the 6 GSLMRGs with immune cell infiltration and inflammatory factor expression, respectively.

### Consensus clustering

We performed consensus clustering by k-means method to identify different subtypes associated with GSLMRGs expression using “ConsensusClusterPlus” R package.

### Functional enrichment analysis

The Gene Ontology (GO) and the Kyoto Encyclopedia of Genes and Genomes (KEGG) functional enrichment analysis were performed for the DEGs using the “clusterProfifiler”, “circlize”, and “fgsea” R package. And Proteomap (https://www.proteomaps.net/) was applied to analyze the functional categories of DEGs.

### Single-cell RNA statistical processing

The “seurat” R package was used to create seurat objects from scRNA-seq data, and cells were normalized and scaled.29608179 We filtered cells by the “seurat” R package based on the following exclusion criteria: 1) cells with less than 200 genes expression. 2) cells with >5000 genes expression. 3) cells having >10% mitochondrial gene content. We retained a total of 43,910 cells. The number of principal components (PCs) was set to 15 for subsequent dimensional clustering, and the “harmony” function 31740819 was used to integrate the samples and remove batch effects. The unsupervised cell clusters based on the top 15 PCA principles were acquired using the graph-based cluster method (resolution = 0.5). Cell clusters were visualized by t-distributed stochastic neighbor embedding (tsne) plot. The “FindAllMarkers” function of the Wilcoxon rank-sum test algorithm was used to calculate the marker gene for each cell cluster under the following conditions: 1) logFC >0.25; 2) P<0.05; 3) minimum percentage (min.pct) >0.1. For detailed identification of fibroblast clusters, clusters of fibroblasts were selected by using re-tSNE analysis, graph-based clustering, and marker gene analysis. Furthermore, the “AddModuleScore” function was used to calculate the score of GSL metabolism pathways in each cell for the re-clustered fibroblasts, and the cells were divided into three groups of high-, medium- and low-GSL metabolism by quartiles.

### Pseudotime analysis

Pseudotime analysis is a cell fate analysis method, the single-cell trajectory analysis was conducted using Monocle2 algorithm (http://cole-trapnell-lab.github.io/monocle-release) (14). The “sample” function was used to randomly select 4000 cells for the subsequent pseudotime analysis. Then we used the “DDRTree” method to reduce the dimensionality of the cell, and then utilized the “reduceDimension” function to calculate the type of cell differentiation state. Finally, we used the “plot cell trajectory” function to display the graph of cell differentiation trajectory. We also applied the “plot pseudotime heatmap” to visualize the change of GSLMRG expression with cell differentiation trajectory (adj p-value<0.05).

### Cell-Cell communication analysis

To enable a comprehensive analysis of intercellular communication molecules, the authors applied the cell-cell communication analysis by “CellChat” R package, a recently developed tool that generates and plots cell-cell communication probabilities and interaction strengths from single-cell transcriptomic data. The normalized count and cell types by Seurat were used for this analysis.

## Results

### Sample data processing

**Figure 1:**
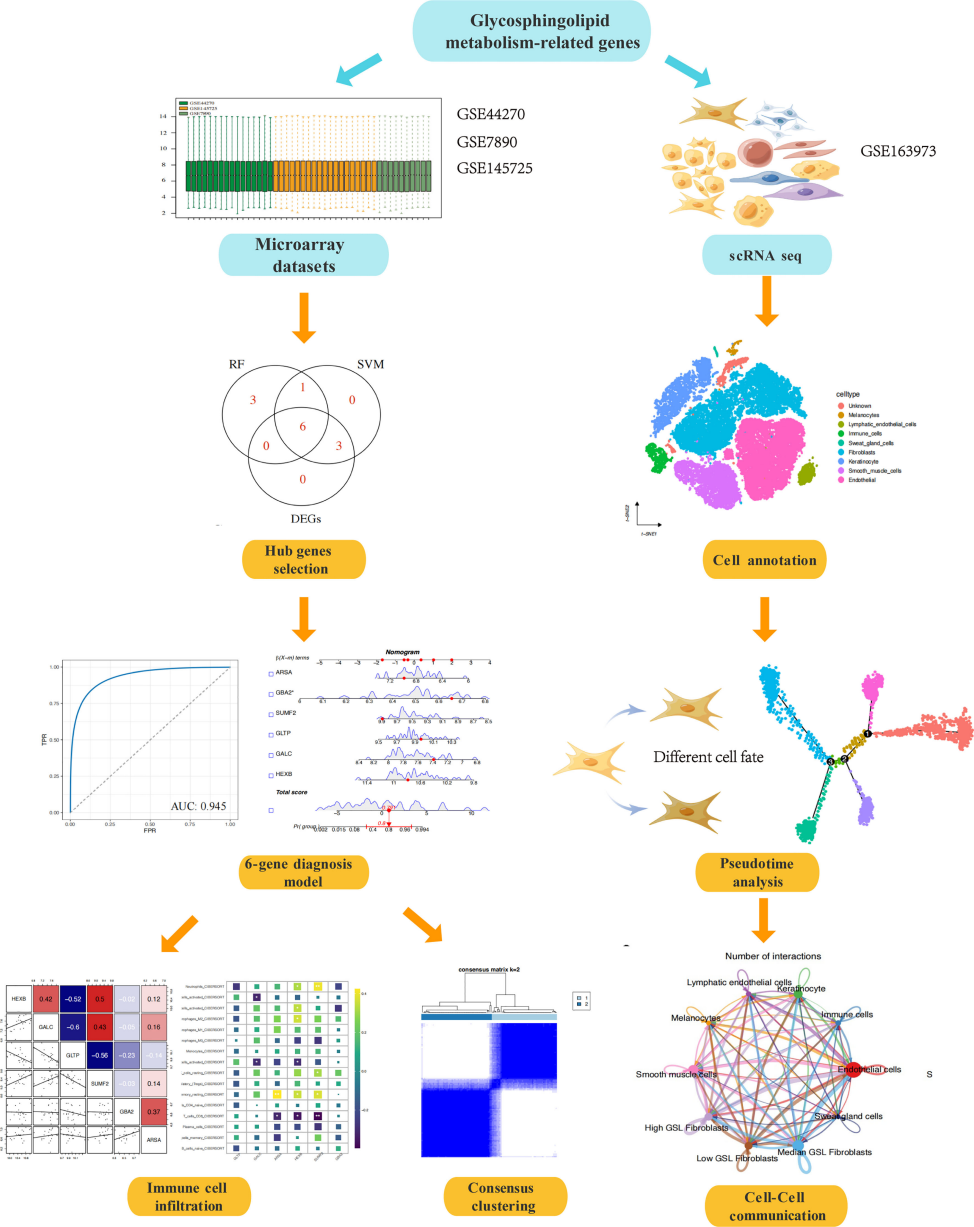
The study workflow.

**Figure 2:**
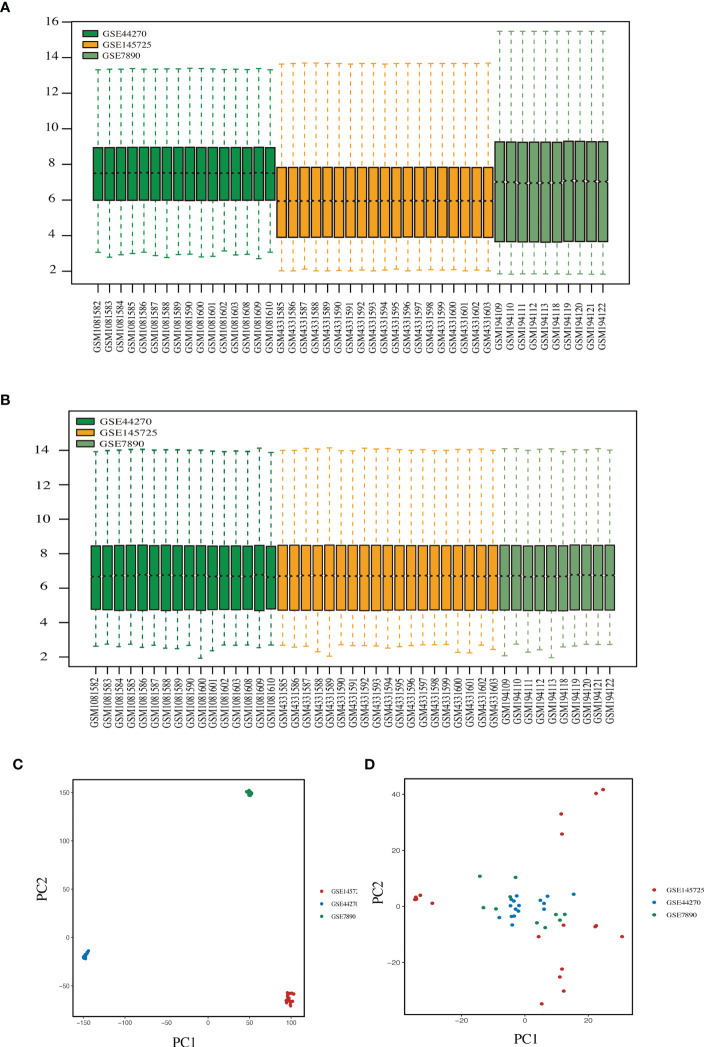
Combining different datasets. **(A, B)** Boxplots of mRNA expression distribution before and after removing batch effects. **(C, D)** PCA plots before and after removing batch effects.

**Figure 1** illustrated the flow of our study. We integrated the expression profiles of the three datasets, as samples from different dataset sources usually have severe batch effects. To eliminate the batch effects among different datasets, ‘ComBat’-based batch effects adjustment was performed. The results for keloid samples and normal samples expression distribution before and after adjustment were illustrated by boxplots (**Figures 2A, B**) and PCA plots (**Figures 2C, D**), respectively. Finally, we obtained a comprehensive dataset of 23 keloid and 22 normal samples after integration, removing batch effects, and normalization.

### Identification of diagnostic markers

**Figure 3:**
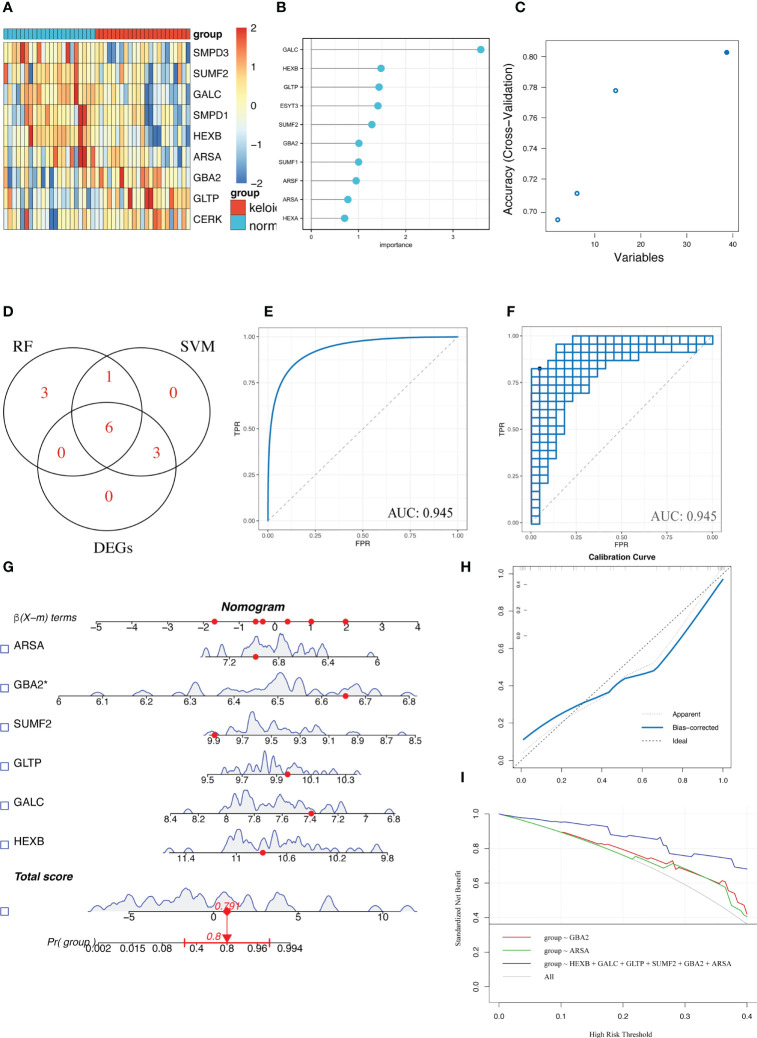
Diagnostic model of keloid was constructed and evaluated. **(A)** Heatmap for differential analysis of GSLERGs between keloid and normal samples. **(B)** Random forest algorithm screening for gene importance ranking. **(C)** SVM-REF algorithm screening for genes. **(D)** Venn plot showed the intersection genes of the top 10 of RF, SVM-REF and DEGs. **(E)** ROC curves under AUC values in the diagnostic model built using the 6 GSLERG. **(F)** Bootstrap resampling algorithm to validate the model. **(G)** Keloid prediction by nomogram. **(H)** Calibration curve to evaluate the nomogram. **(I)** Decision curves to assess the predictive performance of the model.

Differential gene analysis was performed to determine the DE-GSLMRG between keloid and normal samples to obtain relevant differentially expressed genes(p-value<0.05) and finally obtained 9 genes, and we could see the distribution of differential gene expression in each sample by heatmap (**Figure 3A**). To further precisely obtain the key genes, we entered 46 GSLMRGs into the RF classifier and we calculated the gene importance and visualized the top ten genes (**Figure 3B**). In addition, we detected 39 genes as diagnostic markers with the highest accuracy when modeled by the SVM-RFE algorithm (**Figure 3C**). We took their respective TOP10 through importance rankings predicted by the two machine learning algorithms and intersected them with 9 DEGs, and finally the common 6 intersected genes (ARSA, GBA2, SUMF2, GLTP, GALC, HEXB) were used as the final diagnostic markers (**Figure 3D**), and the 6-GSLMRG diagnostic model were constructed by multifactorial logistic regression, which was evaluated by using the receiver operating characteristic (ROC) curve and areas under the curve (AUC), and the AUC value was 0.945 (**Figure 3E**). The model was re-sampled 1000 times by bootstrap algorithm to verify the stability and accuracy of the model, and the mean AUC value was 0.919 with 95% confidence interval (CI) of 0.864-0.943, which all indicated the high accuracy of our 6-GSLMRG model (**Figure 3F**). We further constructed the nomogram (**Figure 3G**), and the efficacy of nomogram was evaluated by calibration plot (**Figure 3H**) and decision curve (**Figure 3I**), respectively, demonstrating the high accuracy and sensitivity of the nomogram.

### Immune infiltration analysis

**Figure 4:**
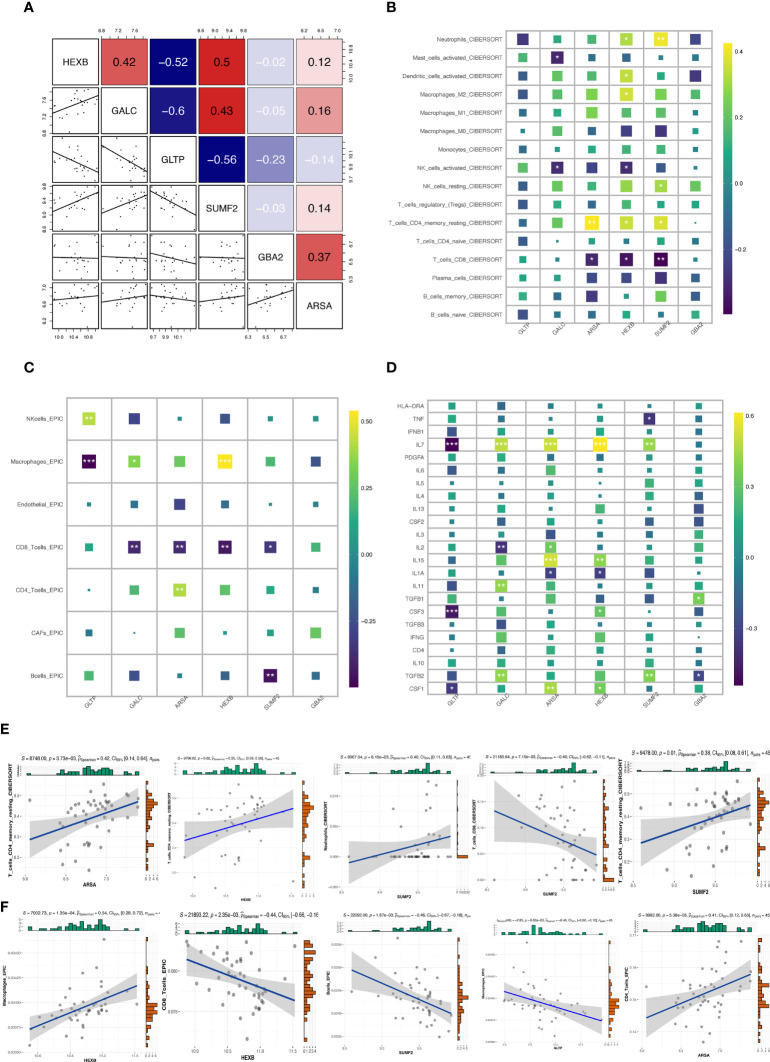
Correlation between candidate genes and immune cell infiltration. **(A)** Co-expression patterns of 6 GSMRG in all samples based on the Pearson correlation analysis. **(B, C)** Heatmap of correlation between 6 GSMRG and immune cell infiltration in Cibersort and EPIC algorithm. **(D)** Heatmap of correlation between GSMRG and inflammatory factors. **(E–F)** Top 5 correlation plots in Cibersort and EPIC algorithm. *p < 0.05, **p < 0.01, ***p < 0.001.

To investigate the co-expression relationship between these six key genes to predict their intrinsic possible regulatory mechanisms, we used Pearson correlation analysis to visualize their co-expression relationship (**Figure 4A**), in which some genes were significantly correlated. For example, GALC and GLTP had the highest negative correlation coefficient of -0.6, and HEXB and SUMF2 had the highest positive correlation coefficient of 0.5. In addition, we performed immune cell infiltration analysis on keloid and normal tissue samples. We calculated the degree of immune cell infiltration in the samples by two immune infiltration algorithms (CIBERSORT and EPIC) and correlated 6 GSLMRGs with the predicted degree of immune cell infiltration and the currently known inflammatory factor expression distribution in the samples (**Figures 4B–D**), where T cells were more strongly correlated with 6 GSMRG. We found that CD8+ T cells showed significantly negative correlation with GALC, ARSA, HEXB, and SUMF2, while CD4+ T cells showed significantly positive correlation with ARAS. Macrophages showed significantly negative correlation with GLTP and positive correlation with HEXB. NK cells showed positive correlation with GLTP and negative correlation with GALC and HEXB. Further, we visualized the top5 correlation plots between GSMRG and the corresponding immune cells in the CIBERSORT and EPIC algorithm, respectively (**Figures 4E, F**).

### GSLMRG-based keloid classification

**Figure 5:**
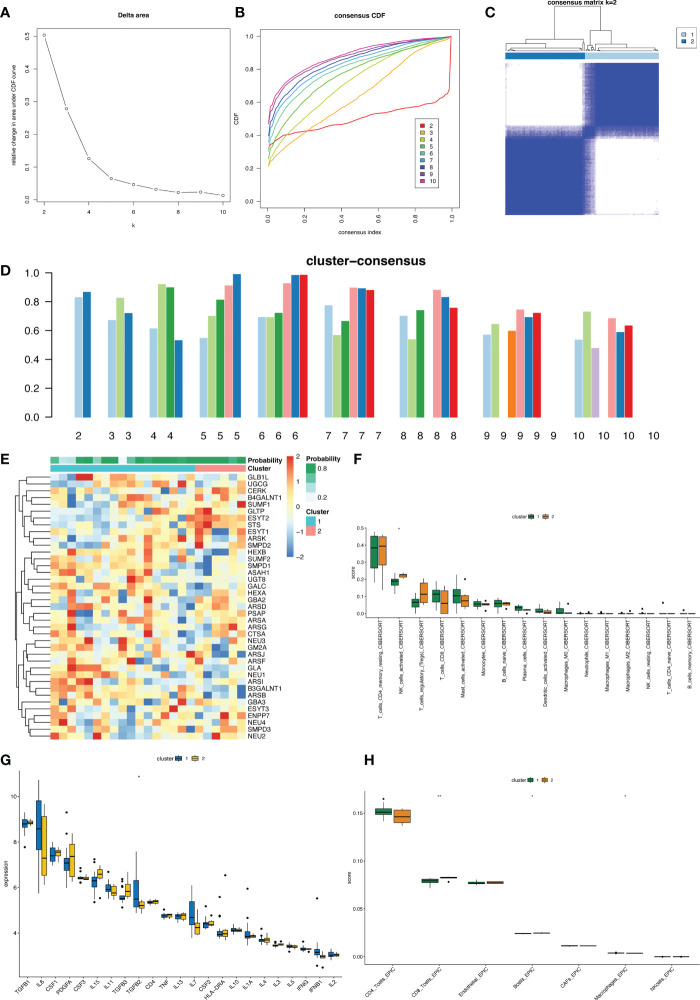
Unsupervised clustering analysis in keloid. **(A)** The empirical cumulative distribution function (CDF) plots revealed the consensus distributions for each k. **(B)** The area change under CDF curve when k=2-10. **(C)** The circular manhattan (CM) plot exhibited the clusters at k = 2. **(D)** The bar plot showed the score of each subtype for the number of clusters k from 2 to 10. **(E)** Heat map showing the distribution of GSLMRG expression between different clusters. **(F–H)** Box plots of the distribution of immune cell infiltration and inflammatory factors expression in different clusters.

Next, we use k-means cluster analysis to classify keloids into groups. The categorical variable k was increased from 2 to 10, and we found that the lowest correlation between groups and the highest correlation within groups when k=2. Therefore, the 23 keloid samples could be classified into two clusters based on the expression of genes related to glycosphingolipid metabolism (**Figures 5A–D**). We visualized the distribution of GSMRG expression between subtypes by heatmap (**Figure 5D**) and further compared the immune cell infiltration and the expression ofinflammatory factors in these two clusters (**Figures 5E–H**).

### Enrichment analysis

**Figure 6:**
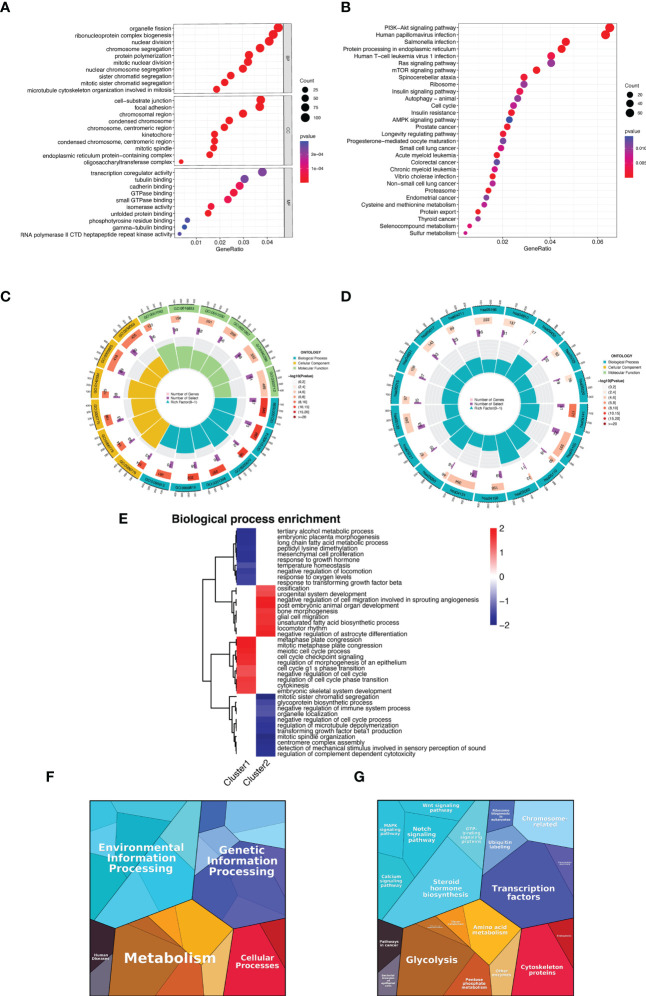
Functional enrichment analysis. **(A, C)** GO enrichment analysis of DEGs between fibroblasts of different subtypes. **(B, D)** KEGG enrichment analysis of DEGs between fibroblasts of different subtypes. **(E)** GSEA enrichment analysis of biological functions between two clusters. **(F, G)** Functional categories of differentially expressed genes between fibroblasts of different subtypes.

In order to explore the functional and pathway differences between subtypes and reveal the potential mechanisms of disease progression between subtypes, we used pathway enrichment analysis based on the differential genes between the two subtypes, and the results of GO enrichment analysis showed that DEGs from the two subtypes were enriched in organelle fission, ribonucleoprotein complex biogenesis, nuclear division, and other biological functions (**Figures 6A, C** and **Supplementary Table S2**). Further, we analyzed the specific biological functions of two subtypes by GSEA enrichment analysis, the results showed that Cluster 1 is mainly enriched in metaphase plate congression, meiotic cell cycle process, cell cycle checkpoint signaling pathway, etc. Cluster 2 is mainly enriched in ossification, urogenital system development, bone morphogenesis pathway, etc. (**Figure 6E** and **Supplementary Table S3**). KEGG enrichment analysis showed that DEGs were mainly enriched in PI3K-Akt signaling pathway, Ras signaling pathway, mTOR signaling pathway, autophagy, cell cycle, and other pathways (**Figures 6B, D**). Then, protein map pathway analysis was performed to classify their functions. It was found that these genes were enriched in five pathways, namely Environmental Information Processing, Genetic Information Processing, Human Disease, Metabolism, and Cellular Processes (**Figure 6F**), mostly in transcription factors, steroid hormone biosynthesis, glycolysis, cytoskeleton proteins, notch signaling pathway, and amino acid metabolism (**Figure 6G**), which proved to be highly relevant to the occurrence, development, and treatment of keloids. All these results demonstrated the application value of GSLMRG-based keloid staging and provided potential mechanisms for how these GSLMRGs influence keloid progression.

### Analysis of high cellular heterogeneity in human keloid tissues by single-cell RNA-seq profiling

**Figure 7:**
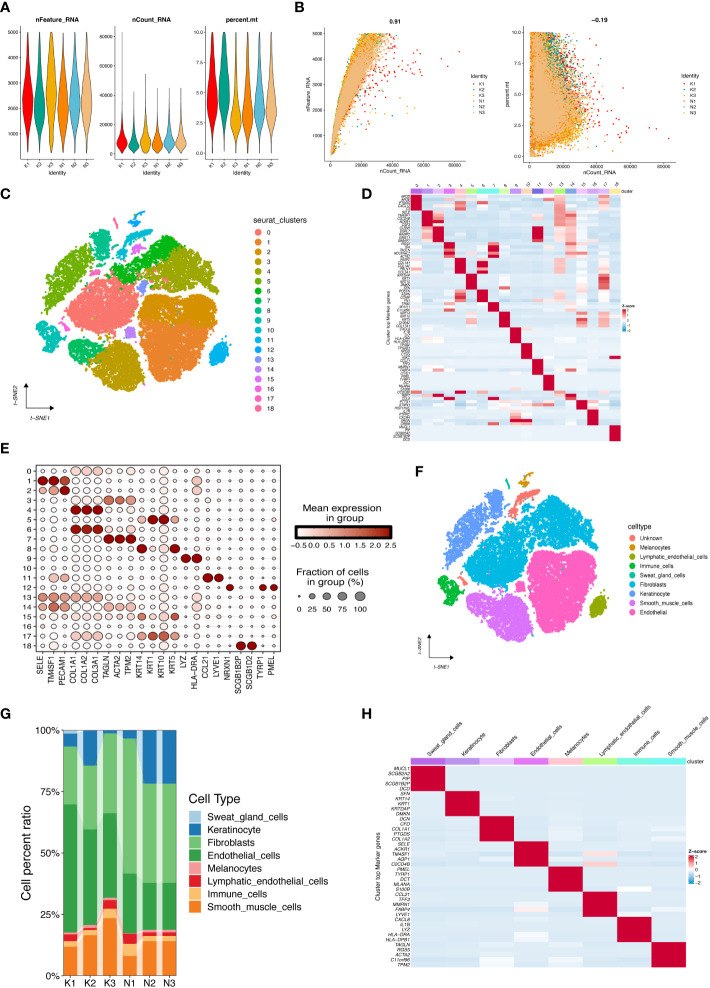
Cell populations and marker genes in keloid and normal skin. **(A)** After standard quality control of all cells from three keloids and three normal tissues, 43,910 cells were included in the analysis. **(B)** The number of genes detected was significantly correlated with the sequencing depth, with a Pearson correlation coefficient of 0.91; the same number of mitochondria was detected at different sequencing depths. **(C) **The cell clusters visualized by the dimensional reduction of t-distributed stochastic neighbor embedding (t-SNE). **(D)** Heatmap showing the top 5 genes per cell cluster after differential analysis to obtain marker genes. **(E)** Dot plot showed annotation of cell clusters by known markers. **(F)** tSNE plot presented cell type annotation for each cluster. **(G)** Proportions of distinct cell types for different samples. **(H)** Heatmap showed the top 5 marker genes between cell types.

We used the scRNA-seq data of three normal and three keloid tissues from the GEO database to reveal the inherent cellular heterogeneity of skin tissues. After strict quality control, we excluded cells of lower quality and selected a total of 43,910 cells for subsequent analysis (**Figure 7A**), with a mitochondrial UMI rate of less than 10% per cell, and detected a significant correlation between gene number and sequencing depth (**Figure 7B**). We identified 19 cell clusters by performing PCA dimensionality reduction using the first 15 principles and setting a resolution value of 0.5. Different cell populations exhibited high heterogeneity (**Figures 7C, D**). We identified detailed cell types based on marker genes from previous studies (15), and 8 categories were annotated (**Figures 7E, F, H**), including melanocytes (cluster 12, marker genes are TYRP1, PMEL), lymphatic endothelial cells (cluster 11, marker CCL12, LYVE1), immune cells (cluster 9, marker genes are LYZ, HLA-DRA), sweat gland cells (cluster 18, marker genes are SCGB1B2P, SCGB1D2), fibroblasts (clusters 0,4,6,13,15, marker genes are COL1A1, COL1A2 COL3A1), keratinocyte (clusters 5, 8, 17, marker genes are KRT14, KRT1, KRT10, KRT5), smooth muscle cells (clusters 3, 7, marker genes are TAGLN, ACTA2, TPM2), endothelial cells (clusters 1, 2, 14, marker genes are SELE, TM4SF1, PECAM1). **Figure 7G** also showed the proportion of cells in each sample.

### Pseudotime analysis revealed changes in glycosphingolipid metabolism pathway activity during fibroblast differentiation

**Figure 8:**
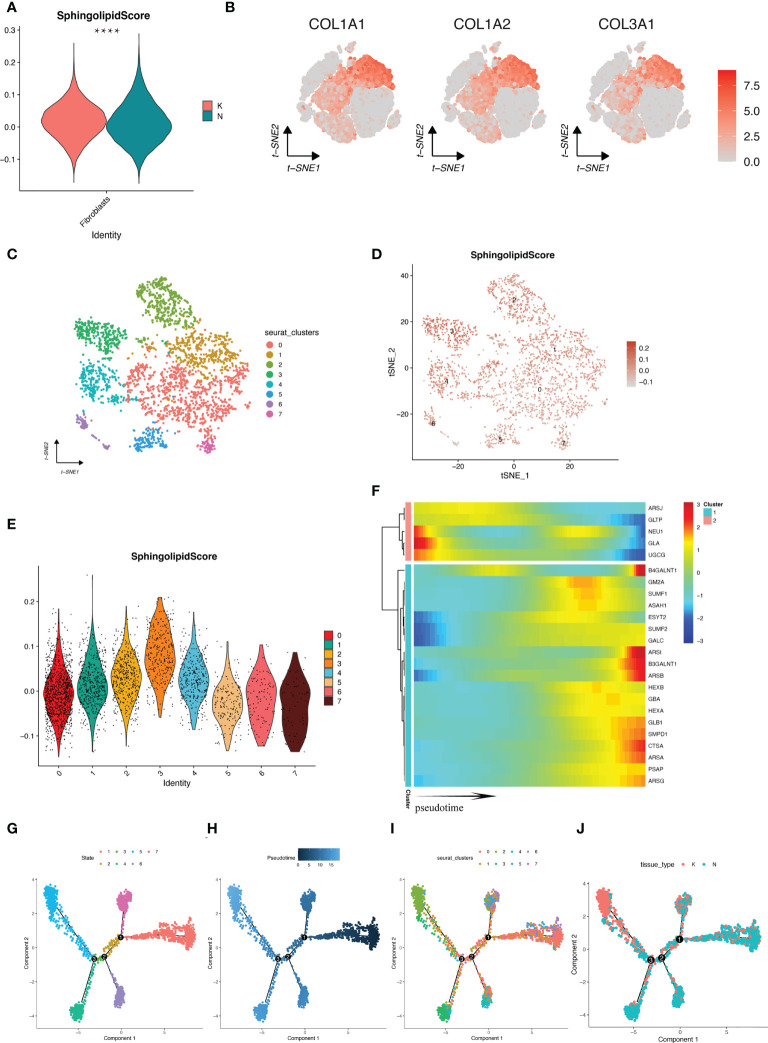
Progression of fibroblast cell profiles revealed by pseudotime analysis. **(A)** GSL metabolic pathway scores of fibroblasts in keloid and NS. **(B)** tSNE plots showed the expression of marker genes in fibroblasts. **(C)** Fibroblasts were clustered again by downscaling and shown by tSNE plots. **(D)** tSNE plots of the GSL metabolic pathway scores of individual fibroblasts. **(E)** The GSL metabolic pathway scores of the fibroblast subpopulations that were downscaled again. **(F)** Heatmap showing the expression changes of genes in GSL metabolic pathway with fibroblast differentiation. **(G–J)** Trajectory differentiation maps according to cell differentiation status, cell development time coloring, cell cluster and tissue type. ****p < 0.0001.

Due to the significant difference in GSL metabolism pathway activity in fibroblasts between keloid and normal tissues (**Figure 8A**), fibroblasts were considered to be the focus cell population in this study. All fibroblasts were highly expressed with marker genes COL1A1, COL1A2, COL3A1 (**Figure 8B**). We then further extracted the transcriptome data of fibroblasts and reanalyzed with tsne visualization (**Figure 8C**). We scored the activity of GSL metabolism pathway in each cell by “AddModuleScore” function in fibroblasts of different subtypes and displayed them in **Figures 8D, E**, in which cluster 3 had the highest activity and cluster 7 had the lowest activity. To further investigate the detailed cell trajectory of fibroblasts, we performed pseudotime analysis. **Figure 8G** indicates that there are seven states during fibroblast differentiation, marked by different colors. **Figure 8H** indicates that the darker the blue color, the earlier the cells differentiate, indicating that fibroblasts differentiate from right to left over time, with the lightest blue color being the most recently differentiated cells, and cluster2 is the latest differentiated fibroblast. **Figure 8I** shows how cell subpopulations evolves and differentiates from each other and **Figure 8J** displays the distribution of keloid and normal skin fibroblasts during the differentiation process. In keloid tissues, due to the increase of myofibroblasts and stromal fibroblasts, we further analyzed the expression of ADAM12 and a-SMA (encoded by ACTA2) in fibroblasts. **Supplementary Figure S1** showed that both genes were relatively highly expressed in cluster2, indicating that cluster2, as the terminal stage of fibroblast differentiation, almost exclusively consisted of myofibroblasts and stromal fibroblasts in keloid tissues.We also showed the expression changes of GSL metabolism genes during the differentiation of these fibroblasts, **Figure 8F** visualized the heatmap of GSL metabolism genes that changed accompanying with the differentiation of fibroblasts. These genes are grouped into 2 types, where their expression increases or decreases with cell differentiation, respectively, indicating these genes may exert different functions in the pathogenesis of fibroblast differentiation induction and influence the activity of the GSL metabolism pathway.

### Cell-cell communications

**Figure 9:**
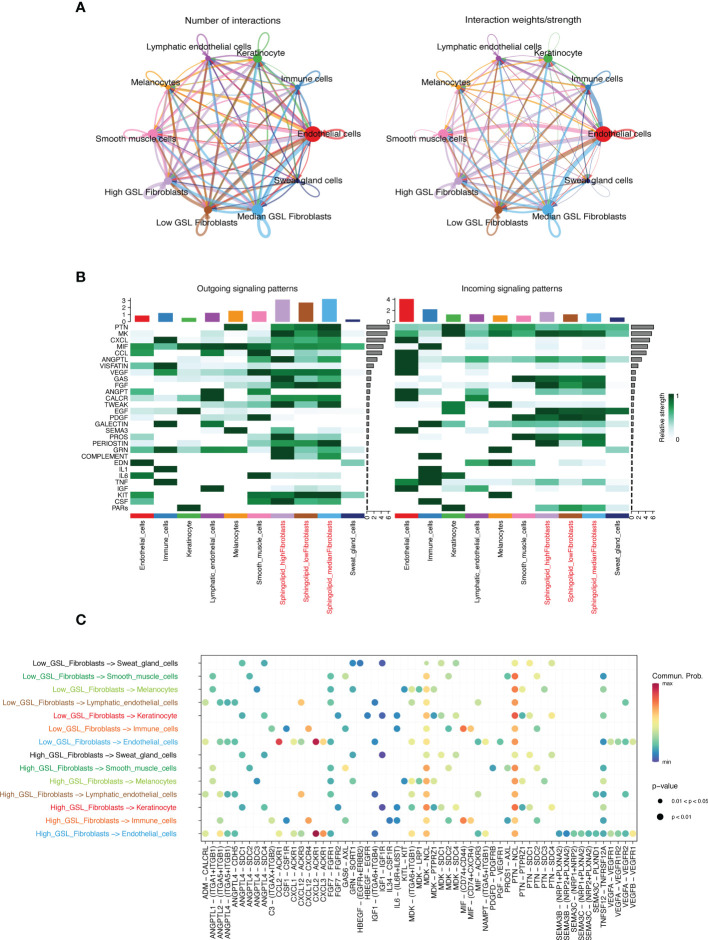
**(A)** Integrated cell-cell communication networks drawn by number and weight of interactions. **(B)** The heatmap of outgoing/incoming interaction strength for 10 cell types. **(C)** The dot plot of outgoing and incoming interaction signal pathways for fibroblasts of two subtypes.

To decipher intercellular signaling, we used the “CellChat” R package to perform cell-cell communication analysis between different cell types. We classified fibroblasts into three types of high-, median-, low-GSL metabolism activity based on quartiles bounded by 25% and 75% of previous scores. The aggregated cell-cell communication networks were constructed by interaction numbers (**Figure 9A**) and interaction weights (**Figure 9B**). The interaction strengths of cell incoming and outgoing signaling were plotted in **Figure 9C**, which indicated that fibroblasts play a key role in intercellular communication. Fibroblasts with low GSL metabolism activity had lower strengths in both incoming and outgoing signaling pathways than the other two types of fibroblasts. We further investigated the signaling sources of 2 types of cells (high-GSL metabolism activity fibroblasts and low-GSL metabolism activity fibroblasts) and we analyzed the different incoming and outgoing signaling pathways of the two types of fibroblasts based on the relative expression of ligand-receptor (L-R) pairs (**Figure 9C**), and we compared the intercommunication between the two types of fibroblasts and other cells, and the high-GSL metabolism activity fibroblast could additionally communicate cellularly with smooth muscle cells through PDGFD-PDGFRB interaction, with keratin-forming cells through ITGA6-ITGB1 interaction, with endothelial cells through SEMA3B signaling pathway, and with endothelial cells through ITGA5-ITGB1, with immune cells through IL34-CSF1R, PROS1-AXL, TNFSF12-TNFRSF12A interaction, the fibroblasts with high-GSL metabolism activity can additionally communicate with keratinocytes *via* HBEGF-EGFR interaction, with endothelial cells *via* PROS1-AXL interaction, with sweat gland cells *via* EGFR-ERBB2 interaction, with lymphatic endothelial cells *via* SEMA3C- PLXND1 interaction, suggesting that the levels of GSL metabolism pathway activity in fibroblasts may affect other cell types through these receptors.

## Discussion

Keloid is a fibrous tissue hyperplastic disease after trauma and inflammatory stimulation of the skin, characterized by fibroblast proliferation and collagen deposition (16, 17). keloid causes patients not only serious cosmetic problems, but also functional traits such as pruritus and pain around the lesion and lesions, which seriously affect the quality of life of patients (18). Currently, the efficacy of conventional treatment on keloid is limited. Therefore, exploring the potential new mechanisms and new biomarkers may benefit the treatment of keloid and improve the prognosis of keloid.

SL serves as one of the major components of eukaryotic lipids, its metabolism in the skin is currently receiving increasing attention. Recent studies showed that SL controls the heterogeneity of dermal fibroblasts and that GSL, a subtype of SL, is involved in determining the developmental differentiation of cells (10, 19). However, GSL metabolism pathway have been less well studied in the skin, and by combining microarray datasets and Single-cell RNA-seq, our study was the first comprehensive analysis to reveal the role of GSL metabolism in the development of keloid. We constructed a keloid diagnostic model using GSLMRGs. In addition, this study also investigated the role of GSL metabolism pathway in cell differentiation and communication.

In this study, the differential GSLMRGs of keloid and the top ten genes with the highest importance from machine learning algorithms Random Forest and SVM-RFE were intersected, and six candidate GSLMRGs were identified: ARSA, GBA2, SUMF2, GLTP, GALC, and HEXB. GSL can play a regulatory role in the airway of lung inflammatory fibrotic diseases, such as Cystic fibrosis, and inhibition of GBA2 can control the role of Cystic fibrosis inflammatory response (20). SUMF2 is a member of the formylglycine-generating enzyme family and may mediate airway inflammation in allergic asthma by regulating IL-13 expression (21). GLTP is a small (24 kD) amphipathic protein. They have been shown to be involved in the non-vesicular transport of various SLs. In addition, their potential functions such as drug resistance, differentiation, neurodegeneration, surface adhesion, and apoptosis have been reported (22). High GALC expression can regulate migration during tumor growth by regulating senescent fibroblasts in tumors (23). And the higher expression of HEXB is associated with poor prognosis in glioblastoma patients. But none of these genes have been studied in keloid, implying their great research value in keloid. By constructing a diagnostic model of keloid by multi-factor logistic regression of these six genes, we could find high AUC value under the ROC curve and evaluated the model by bootstrap resampling method, indicating that our diagnostic model constructed by GSLMRG has high diagnostic accuracy.

Since multiple immune cells and inflammatory factors are involved in the formation and development of keloid 28108895, we applied the CIBERSORT, EPIC algorithm to generate immune cell expression profiles of keloid. Previous study showed that reducing CD8+ T cells may serve as a biomarker and therapeutic method for keloids (24). Based on this, we calculated the correlation between the six diagnostic genes and the degree of immune cell infiltration and found that CD8+ T cells were correlated with GALC,ARSA,HEXB and SUMF2, CD4+ T cells were correlated with ARSA, implying that GSL metabolism pathway may impact on the growth and development of keloid through regulating immune T cells. IL-7 is involved in ECM production by exogenous TGF-β1-activated subconjunctival fibroblasts, suggesting that IL-7 administration could be a novel therapeutic target to prevent undesirable bleb scar formation during post-surgical healing (25). A strong correlation between IL-7 and GLTP, GALC, ARSA, HEXB, and SUMF2 was found, suggesting that IL-7-based inflammatory factors may involve in keloid growth and development through associating with the GSL metabolism pathway. Revealing the important role of the GSL metabolism pathway may facilitate keloid immunotherapy.

Because of the presence of heterogeneity in keloids, the curative effects of the available clinical treatments are often unsatisfactory (2). To achieve the precise treatment for keloids, we divided keloids into two clusters by GSLMRGs expression,We compared the differences between the two groups in terms of immune infiltrating cells, inflammatory factors, and explored the enrichment pathways between the two groups. It has been documented that macrophages promote collagen production and angiogenesis to accelerate wound healing. The occurrence of keloids in different parts of the body is associated with the number and subtype of macrophages (26). NK cell is an important component of the innate immune system and may be involved in keloid formation. Overexpression of Smad7 inhibits NK cell in keloids proliferation and migration (27). B cells were shown to be significantly upregulated in keloids compared to normal skin (28). TGFB2 was shown to play an important role in the development of fibrotic disease, and inhibition of TGFB2 attenuated fibrosis and inflammation (29). CD8 T cells, macrophages, NK cells, B cells, and TGFB2 expression were significantly different in two subtypes of keloids (P-value<0.05), demonstrating that they could be studied in depth as important targets for treatment.

We identified a total of 19 cell clusters by the harmony integration algorithm (based on Seurat v4) to eliminate batch effects between multiple samples, with 5 cell clusters in Fibroblasts, 3 cell clusters in Endothelial cells, 2 cell clusters in Smooth muscle cells Keratinocyte 3 clusters, 1 cell cluster in Immune cells, 1 cell cluster in Lymphatic endothelial cells, 1 cell cluster in Melanocytes, of which 2 cell clusters are unknown. Since studies have demonstrated that fibroblasts play an important role in the development of keloids, our next study focused on fibroblasts (30).

We further divided fibroblasts into 8 cell clusters and calculated the GSL metabolic pathway activity for each cell cluster. It has been found that the SL metabolic pathway regulates the heterogeneity of dermal fibroblasts, resulting in phenotypic alterations in fibroblasts of different subtypes (19). We further investigate whether a similar effect of the GSL metabolic pathway exists for dermal fibroblasts. In the cell differentiation trajectory, fibroblast differentiation ended with two different cell fates and overall GSL metabolism pathway activity increased with the cell differentiation trajectory, which suggest that the GSL metabolic pathway may have involved in the differentiation and phenotypic function regulation of dermal fibroblasts. Among them, the expression of ARSJ, GLTP, GLA, NEU1, and UGCG decreased with the differentiation of fibroblasts, while the expression of SUMF2, GALC, HEXB, and ARSA increased with the differentiation trajectory of cells. These findings suggest that these GSLMRGs can be divided into two classes with potentially opposite roles in fibroblast differentiation trajectories. Then these two classes of genes may function in balancing with each other and result in different cellular functions and cell fates once the state is disrupted, which provides a basis for our subsequent treatment of keloids by regulating GSL metabolism mechanisms. However, more experiments are needed to validate the hypothesis.

We then compared the cell-cell communication between the two subtypes of fibroblasts and cells of other types. Fibroblasts with high GSL metabolism pathway activity can communicate with smooth muscle cells through PDGFD-PDGFRB interaction, which functions in fibrosis and neovascular formation (31). Besides, fibroblasts with high GSL metabolism pathway activity can also communicate with keratinocytes through ITGA6-ITGB1 interaction, which play a role to promote cancer cell invasion and metastasis in a variety of cancers, such as cholangiocarcinoma and triple-negative breast cancer (32, 33). In addition, the fibroblasts with high GSL metabolism pathway activity can communicate with endothelial cells through the SEMA3B signaling pathway, while SEMA3B is known to be an inhibitor of angiogenesis and cell proliferation. Except for these mentioned above, these fibroblasts can communicate with the immune cells through IL34-CSF1R, ITGA5-ITGB1, PROS1-AXL, and TNFSF12-TNFRSF12A interactions as well. Previous study confirmed that IL34 functions in skin during development, therapeutic interventions targeting IL34 and CSF1 may provide satisfactory immunotherapy effects (34). PROS1-AXL is also a key regulator in inflammation and angiogenesis, and TNFSF12-deficient mice exhibit reduced epidermal proliferation (35, 36). Fibroblasts with low GSL metabolism pathway activity can communicate with keratinocytes *via* HBEGF-EGFR interaction, which is activated in many patients with malignancies and can promote skin wound healing (37). Besides, fibroblasts with low GSL metabolism pathway activity can communicate with endothelial cells *via* PROS1-AXL interaction, with sweat gland cells *via* EGFR-ERBB2 interaction, and with Lymphatic endothelial cells *via* SEMA3C-PLXND1 interaction. EGFR-ERBB2 is considered as an anti-cancer target in a variety of cancers, such as breast cancer, malignant peripheral nerve sheath tumors, suggesting that the changes in GSL metabolism pathway activity in fibroblasts may affect cells of other types through these specific ligand-receptor interaction (38). However, more investigations are needed to reveal the exact mechanisms.

In conclusion, we combined the microarray datasets and single-cell analysis to explore the role of GSL metabolism pathways in keloid for the first time, providing new insights into the role of communication between keloid fibroblasts and cells of other types, suggesting potential diagnostic and therapeutic strategies and having important implications for the study of keloid.

## Conclusion

We explored the potential role of GSL metabolism pathway in keloid, classfied keloids based on GSLMRGs expression patterns, provided a set of gene markers including GLTP, GALC, ARSA, HEXB, SUMF2, and GBA2, and constructed a diagnostic model for keloid. We further revealed the alteration of GSL metabolism pathway activity in the differentiation of fibroblasts by single cell analysis and the role of GSL metabolism in cell-cell communication.

## Data availability statement

The original contributions presented in the study are included in the article/**Supplementary Material**. Further inquiries can be directed to the corresponding authors.

## Author contributions

Conception and design: BYS, YZ and HC. Data curation and methodology: YHZ, ZC, YD, and LC. Analysis and interpretation of data: GC and BG. Writing of the manuscript: BYS. Review of the manuscript: ZY and BQS. Study supervision: ZY and BQS. All authors contributed to the article and approved the submitted version.
