# Supplementary material for: Revealing the roles of glycosphingolipid metabolism pathway in the development of keloid: a conjoint analysis of single-cell and machine learning
Source: Front Immunol. 2023 Apr 24;14:1139775. doi: 10.3389/fimmu.2023.1139775 (PMC10164993; doi:10.3389/fimmu.2023.1139775)
Supplement: Supplementary file 1 [file DataSheet_1.docx]

Supplementary Material

# Supplementary Figures

## Supplementary Figures
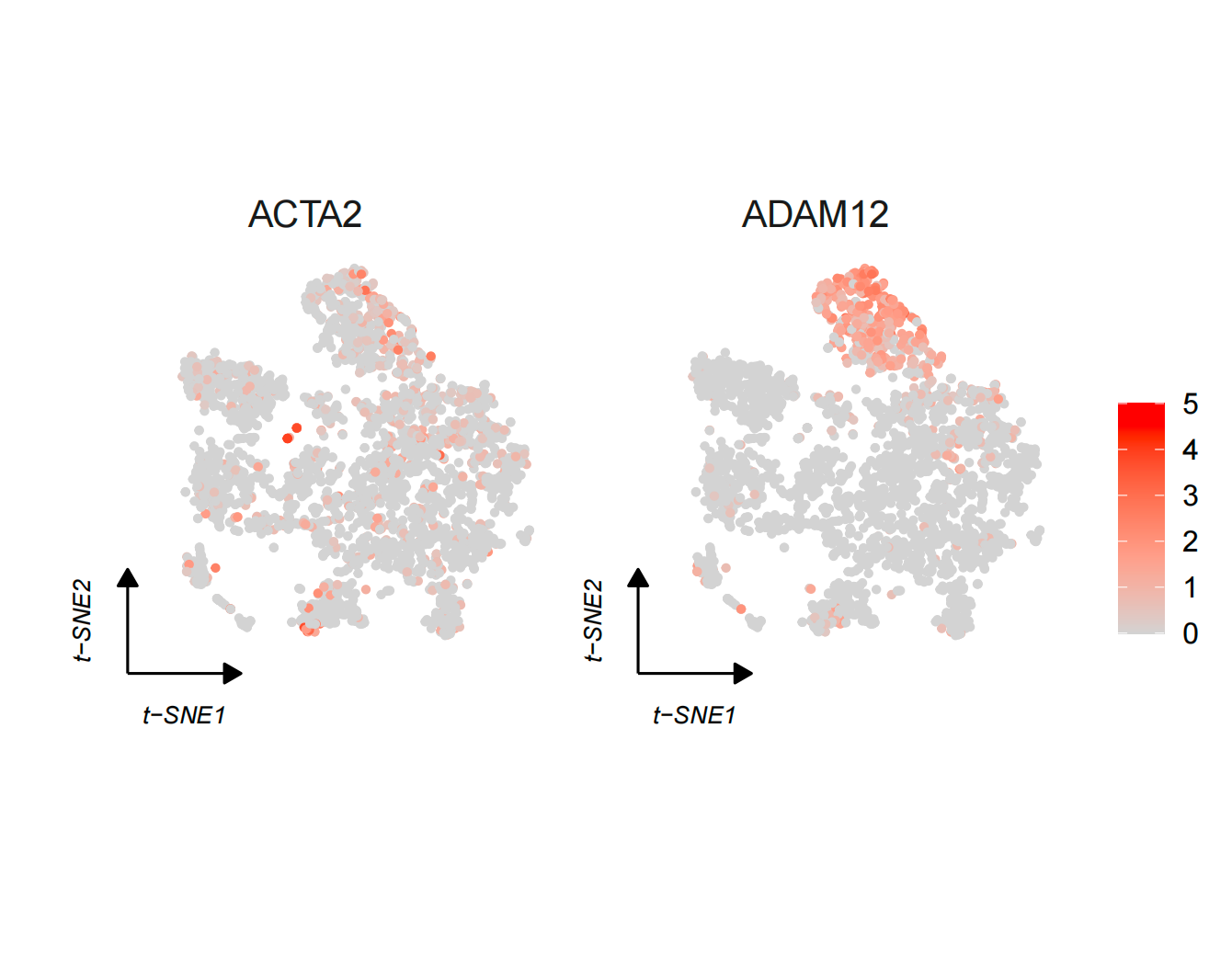
[Supplementary](javascript:;) Figure S1. The expression distribution of ACTA2 and ADAM12 in fibroblasts
